# Synergy of the Bacteriocin AS-48 and Antibiotics against Uropathogenic Enterococci

**DOI:** 10.3390/antibiotics9090567

**Published:** 2020-09-02

**Authors:** Manuel Montalbán-López, Rubén Cebrián, Rosa Galera, Lidia Mingorance, Antonio M. Martín-Platero, Eva Valdivia, Manuel Martínez-Bueno, Mercedes Maqueda

**Affiliations:** Department of Microbiology, Faculty of Sciences, University of Granada, c. Fuentenueva s/n, 18071 Granada, Spain; manuelml@ugr.es (M.M.-L.); rcebrian@ugr.es (R.C.); rosa.galera@uca.es (R.G.); lidiamingo@gmail.com (L.M.); ammartin@ugr.es (A.M.M.-P.); evavm@ugr.es (E.V.); mmartine@ugr.es (M.M.-B.)

**Keywords:** *Enterococcus*, VRE, virulence, antibiotic resistance, antimicrobial peptide

## Abstract

The genus *Enterococcus* comprises a ubiquitous group of Gram-positive bacteria that can cause diverse health care-associated infections. Their genome plasticity enables easy acquisition of virulence factors as well as antibiotic resistances. Urinary tract infections (UTIs) and catheter-associated UTIs are common diseases caused by enterococci. In this study, *Enterococcus* strains isolated from UTIs were characterized, showing that the majority were *E. faecalis* and contained several virulence factors associated to a better colonization of the urinary tract. Their susceptibility against the bacteriocin AS-48 and several antibiotics was tested. AS-48 is a potent circular bacteriocin that causes bacterial death by pore formation in the cell membrane. The interest of this bacteriocin is based on the potent inhibitory activity, the high stability against environmental conditions, and the low toxicity. AS-48 was active at concentrations below 10 mg/L even against antibiotic-resistant strains, whereas these strains showed resistance to, at least, seven of the 20 antibiotics tested. Moreover, the effect of AS-48 combined with antibiotics commonly used to treat UTIs was largely synergistic (with up to 100-fold MIC reduction) and only occasionally additive. These data suggest AS-48 as a potential novel drug to deal with or prevent enterococcal infections.

## 1. Introduction

Urinary tract infections are one of the most common infections in humans. In general, they are uncomplicated infections produced by Gram-negative bacteria [1]. However, in hospital environments, complicated Gram-positive urinary catheter-associated infections caused by *Enterococcus faecium* and *Enterococcus faecalis* have emerged as an important issue [1,2]. In fact, enterococci account for up to 15% of urinary tract infections associated with the use of catheters, with *E. faecalis* being the most prevalent species [3,4]. The genus *Enterococcus* (with 58 described species to date) are ubiquitous, nonsporulating, low G+C content, catalase negative, aerotolerant, Gram-positive bacteria. These bacteria can colonize the gastrointestinal tract and disseminate to the bloodstream under particular conditions. Thus, their role in infectious diseases has evolved from a gut and urinary commensal microorganisms to a major pathogen of concern [5]. Their success as pathogens is related to their survival abilities in a hostile antimicrobial-rich environment, together with the presence of numerous virulence factors and the high genetic plasticity that facilitates the acquisition and transmission of genetic elements that confer antibiotic resistance [6]. Novel developments and strategies to treat and prevent enterococcal infections are required and phage therapy or combined application of known antimicrobial families against enterococci are under study, including bacteriocins as novel drug delivery systems with great potential as potent antimicrobial agents [7,8,9]. Remarkably, *E. faecium* has been included in the World Health Organization priority pathogen list for which appropriate use of new agents is crucial [10]. This scenario has been worsened by multiple factors such as the declining interest of industry in the development of new drugs and the abuse of antibiotic chemotherapy. Several strategies to combat these pathogens are explained in detail elsewhere [11]. Among them, antimicrobial peptides and the combination of existing drugs are promising venues to tackle this problem [12,13].

Bacteriocins are antimicrobial peptides secreted by bacteria. Although they were discovered earlier than penicillin, their development into clinics has been largely delayed due mainly to their proteinaceous nature and sensitivity to physiological conditions (e.g., proteases, ion concentration, or immunogenicity). Remarkably, most bacteriocins target the bacterial cell membrane of sensitive cells using mechanisms of action different from those employed by conventional antibiotics [14]. However, only a few molecules have entered clinical or preclinical trials [14], with information about their toxicity, pharmacokinetics, and pharmacodynamics very scarce. One of the best characterized antimicrobials is the circular bacteriocin AS-48 [15]. It is a cationic 70 amino acids long peptide that is circularized during its maturation process by peptide bond formation between the N- and C-termini, which provides resistance to proteases and stability in a broad range of conditions such as pH, temperature, or salt concentration [16]. AS-48 inserts into the membrane of sensitive bacteria, creating pores in a mechanism that is independent of any receptor [17], thereby reducing the development of stable and transmissible resistance. AS-48 has been well characterized for its activity against food spoilage and pathogenic bacteria and also against diverse human pathogens with a low minimal inhibitory concentration (MIC) in the nano- to micromolar range [18,19,20,21,22,23]. In addition, a potent trypanocidal activity has been recently described [18,20,21]. Moreover, AS-48 has not shown any remarkable toxicity in vivo or in vitro after a preclinical characterization of the molecule, indicating the safety of the molecule for clinical application [24].

Here, we aimed to isolate and characterize urinary tract infection (UTI)-causing enterococci and evaluate AS-48 as a new weapon to combat them. Thus, we assessed the effect of the bacteriocin AS-48 against several genotypically and phenotypically characterized uropathogenic *Enterococcus* strains, including antibiotic-resistant ones. The use of AS-48 alone and in combination with commonly used antibiotics drastically reduced the viability of enterococci in vitro, independently of their characteristics.

## 2. Results

### 2.1. Isolation and Characterization of Uropathogenic Enterococcal Strains

A set of 58 Gram-positive, catalase-negative, and esculin-positive isolated strains (data not shown) associated to human UTIs in men and women (45% and 55%, respectively) were collected in the Hospital Universitario Clínico San Cecilio in Granada (Spain) and genotypically characterized as *Enterococcus*. The strains were initially genotyped by Random Amplified Polimorphic DNA (RAPD) using the M13 primer. The enterococcal strains were classified into 12 genotypes by cluster analysis of the fingerprints based on Pearson’s correlation coefficient, using an 85% similarity as the threshold to delimit each genotype group. Three strains were identified as *E. faecium* (genotypes 1 and 2) by multiplex PCR, while the remaining were identified as *E. faecalis*. Thus, *E. faecalis* showed a much higher prevalence as a UTI-causing species.

### 2.2. Determination of Virulence Factors in the Clinical Isolates

**Figure 1:**
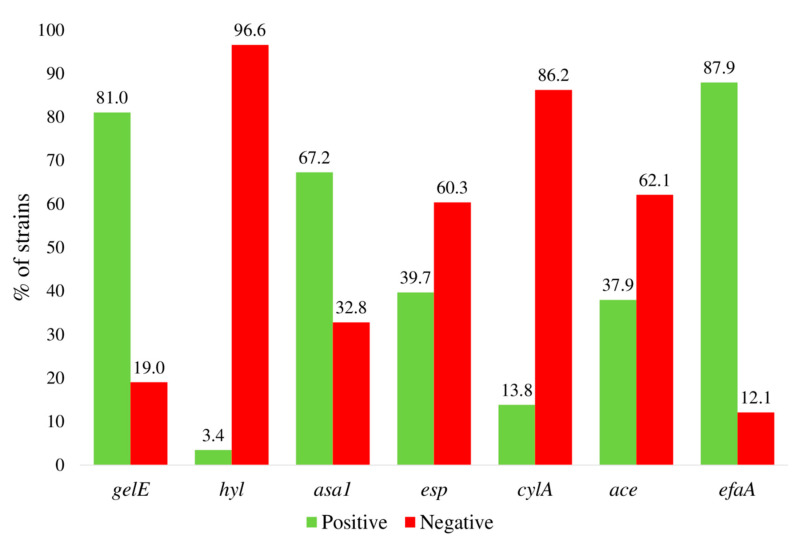
Prevalence of virulence factors among the clinical isolates; *gelE*, gelatinase; *hyl*, hyaluronidase; *asa1*, aggregation substance; *cylA*, cytolysin A; *esp*, extracellular surface protein; *ace*, accessory colonization factor; *efaA*, adhesin.

A set of common virulence factors present in both, *E. faecalis* and *E. faecium*, as well as the occurrence of vancomycin resistance genes (*vanA* and *vanB*) were investigated (Figure 1, Table 1). All the strains were positive to at least one virulence factor: *efaA* (endocarditis antigen A), *gelE* (a matrix metalloproteinase that hydrolyzes gelatin, collagen, and other proteins), or *asa1* (the aggregation substance) genes were the most prevalent, being present in 87.9, 81, and 67.2% of the strains, respectively. Conversely, *hylA* (hyaluronidase) was identified only in one isolate and *cylA* (cytolysin, also called hemolysin) was also absent in 86.2% of the cases (Figure 1). Analysis of co-occurrent virulence factors showed that 6.9%, 19.9%, 27.6%, 27.6%, 13.8%, and 5.2% of the strains were positive for 1, 2, 3, 4, 5, or 6 virulence factors, respectively (Table 1). The most abundant virulence phenotypes observed were the combination of *gelE + asa1 + efaA* (15.52%) and *gelE + efaA* and *gelE + asa1 + esp + efaA* (10.34%), with Esp being an extracellular surface enterococcal protein involved in cell adhesion and biofilm formation [25]. None of the isolates was positive for the vancomycin resistance coding genes *vanA* or *vanB* (data not shown) in agreement with the antibiotic resistance profile (see below). The individual virulence profile for each one of the clinical strains tested is listed in Supplementary Table S1.

### 2.3. Antibiotic Sensitivity of the Clinical Isolates

**Figure 2:**
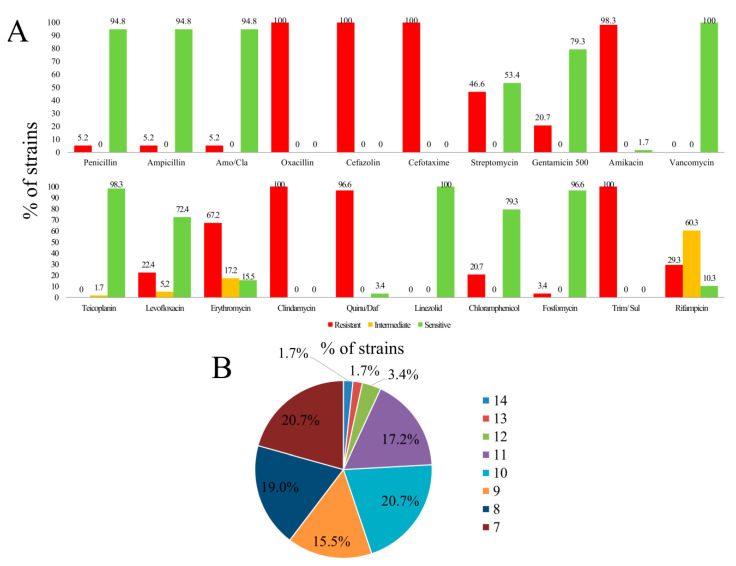
(**A**) Antibiotic resistance incidence in enterococcal isolates from UTIs using WIDER I. (**B**) Percentage of strains simultaneously resistant to a number of antibiotics. Amo/Cla: amoxicillin/clavulanate. Quinu/Daf: quinupristin/dalfopristin. Trim/Sul: trimethoprim/sulfamethoxazole.

All the isolates (n = 58) were tested against a set of 20 antibiotics in a WIDER I panel. All of them were linezolid or vancomycin sensitive (as expected based on the genotypic characterization) and more than 90% were susceptible to penicillin, ampicillin, amoxicillin/clavulanic acid (β-lactams), teicoplanin (glycopeptide), and fosfomycin (phosphonic acid derivative) but resistant to oxacillin (β-lactam), cefazolin (first-generation cephalosporin), cefotaxime (third-generation cephalosporin), clindamycin (lincosamides), and trimethoprim/sulfamethoxazole (sulfonamides, dihydrofolate reductase inhibitors, and combinations) (Figure 2A). Only two were sensitive to quinupristin (streptogramin) and one to amikacin (aminoglycoside). A high prevalence of gentamicin (aminoglycoside), erythromycin (macrolide), and rifampicin (rifamycin) (intermediate) resistance was also detected. With the remaining antibiotics the resistance/sensitivity was variable (Figure 2A). Collectively, all the strains were resistant to at least seven antibiotics and 20.7%, 19%, 15.5%, 20.7%, 17.2%, 3.4%, 1.7%, and 1.7% of the strains were resistant to 7, 8, 9, 10, 11, 12, 13, 14, or 15 different antibiotics, respectively (Figure 2B). Individual data for each one of the strains are available in Supplementary Table S2.

### 2.4. Broth Microdilution MIC Determination

**Figure 3:**
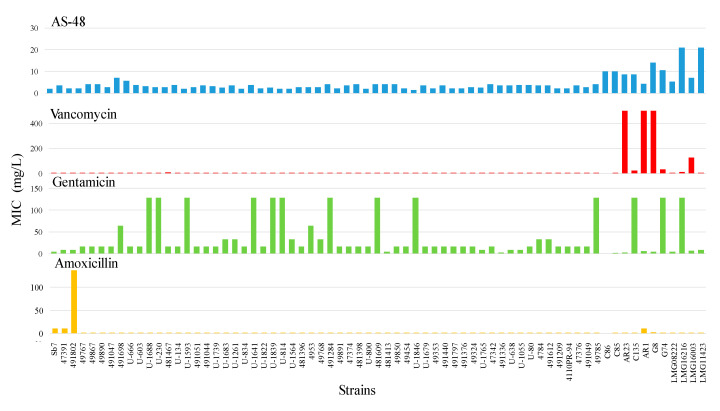
MIC (mg/L) for AS-48 and the antibiotics vancomycin, gentamicin, and amoxicillin/clavulanate for the clinical isolates as well as the lab collection strains.

The available collection of isolated enterococci was tested against AS-48 and four compounds commonly used to treat them (vancomycin, gentamicin, and amoxicillin/clavulanate) (Figure 3, Table 2). To get broader information about the effect of AS-48 on vancomycin-resistant enterococci, 11 strains from our lab collection were also included in this study. AS-48 MIC against uropathogenic enterococci ranged between 1.3–7.1 mg/L with an average of 3.1 ± 1 mg/L, which demonstrates the susceptibility of these clinical strains to AS-48. This average concentration is even below the MIC for the lab enterococci tested, ranging between 5.2–21.0 mg/L (Table 2).

Regarding vancomycin, all the clinical isolates were sensitive to concentrations below 7.8 mg/L with an average MIC of 2.9 ± 1.1 mg/L. Thus, all of them can be considered vancomycin sensitive in the three tests performed (i.e., amplification of resistance-coding genes, WIDER, and broth microdilution assays). As expected, among the lab collection strains the sensitivity was, in general, much lower and some of them showed a vancomycin-resistant phenotype in agreement with the presence of *van* genes previously identified. In particular, the strains AR23, AR1, and G8 were resistant to more than 500 mg/L vancomycin (Table 2). Gentamicin susceptibility was the most variable despite the intrinsic resistance to the aminoglycosides due to poor transport to the interior of the bacteria. The MIC was above 128 mg/L in 10/58 isolates and 3/11 lab strains (Figure 3). Last, the combination of amoxicillin/clavulanate was the most effective against the whole panel. Only three strains (Sb7, 47391, and 491802, all classified as *E. faecium*) showed resistance, in particular, the strain 491802 with 136.7 mg/L. An *E. faecium* strain of the lab collection (*E. faecium* AR1, with a MIC of 10.9 mg/L) also showed increased resistance.

The measured MIC for the four antimicrobials here tested was, in general, not related to each other (Table 2 and Supplementary Table S3), which was expected due to the different mechanisms of action that exist. Remarkably, vancomycin-resistant strains were still sensitive to 4.4–10.5 mg/L AS-48, and this behavior was also observed even in the lab collection strains that displayed a combined resistance to vancomycin and gentamicin (Table 2 and Supplementary Table S3). Similarly, gentamicin resistance was not paired by additional resistance to AS-48 (MIC between 2.8 and 10.5 mg/L for strains resistant to more than 128 mg/L gentamicin) and strains resistant to amoxicillin/clavulanate were sensitive to 1.9–4.4 mg/L AS-48 as well. The individual MIC for each one of the strains and these antimicrobials is listed in Supplementary Table S3.

### 2.5. Synergy between AS-48 and Antibiotics of Clinical Use

#### 2.5.1. Combination of AS-48 and Vancomycin

We selected 12 strains with the highest MICs for vancomycin (above 2.6 mg/L) as well as the lab collection strains to investigate the combined effect with AS-48. In all cases, the calculated fractional inhibitory concentration index (FICI) discarded an antagonistic effect. Interestingly, synergy or additive effects were the most prevalent among the clinical isolates, although an indifferent effect could be observed in five of them (Table 3). These data suggest that there is not a common mechanism of action for the synergy. The most noticeable change was detected in the vancomycin-resistant lab collection strains, where a (partial) synergistic effect was visible even for the most resistant ones (Table 3).

#### 2.5.2. Combination of AS-48 and Gentamicin

The strains with gentamicin MIC > 128 mg/L were studied. As in the previous case, no antagonism was observed. According to the results (Table 4), the effect was, in most cases, additive or indifferent. Synergy was observed only for two clinical and two lab strains. In most cases, the MIC of AS-48 combined with gentamicin remained the same or in a similar order of magnitude. Interestingly, in the cases in which an additive effect was observed, the reduction for the MIC of gentamicin was large (MIC > 128 mg/L for the compound alone and 0.25–2 mg/L in combination with AS-48, i.e., 512- to 64-fold reduction) in comparison with the reduction for the MIC of AS-48 in the combination, which was more discrete or absent (Table 4).

#### 2.5.3. Combination of AS-48 and Amoxicillin/Clavulanate

*E. faecium* species showing MICs above 10 mg/L for amoxicillin/clavulanate were selected to be assayed in combination with AS-48. In two cases, a clear synergistic effect could be observed, including the most resistant strain *E. faecium* 491802 (Table 5). In the other two cases, an additive or no effect was observed. Remarkably, for the strain Sb-7, for which an additive effect was calculated, the MIC of AS-48 remained the same, whereas the antibiotic dropped to 100-fold.

## 3. Discussion

Enterococci are nosocomial pathogens recognized as etiological agents of several diseases (urinary tract infections, endocarditis, and other systemic infections), which exhibit resistance to several groups of antibiotics and easy acquisition of new resistances [9,26]. In this study, we sampled a set of clinical isolates (n = 58) causing UTIs that were phenotypically and genotypically characterized and the results clearly indicate a much higher prevalence of *E. faecalis* as an infectious species (ca. 95%). This is in line with other studies since, usually, 80% of the clinical urinary *Enterococcus* isolates belong to this species [27]. The variety of phenotypes here observed suggests that the strains could have a different origin. Interestingly, the genetic study performed showed no *vanA* or *vanB* genes in the isolates and a heterogeneous distribution of virulence factors. Overall, we observed high prevalence of virulence factors common in UTIs in different geographic areas, i.e., gelatinase (*gelE*) and adhesins (*efaA*, *esp*), that facilitate colonization of urothelial cells, biofilm formation and exchange of genetic material, tissue damage, and resistance to some bioactive peptides [28,29,30,31]. Antibiotic sensitivity was in a similar range as other studies conducted on *E. faecalis* isolated from UTIs, with the highest sensitivity to β-lactams, glycopeptides, linezolid, and fosfomycin and intermediate susceptibility to aminoglycosides or streptomycin [32,33,34]. Remarkably, there was not a correlation between virulence factors and antibiotic resistance among the clinical isolates (Tables S1 and S2). For instance, *E. faecium* 491802 was the only amoxicillin-resistant strain in the collection and displayed only hyaluronidase as a virulence factor. *E. faecalis* U-1688, U-230, and 491284 are gentamicin resistant and contain several virulence factors (*gelE*, *asa1*, *esp*, *efaA*, and also *ace* in the case of 491284), whereas other *E. faecalis* strains with the same virulence factors or even more remained sensitive to vancomycin, gentamicin, and amoxicillin/clavulanate. When the synergy between AS-48 and antibiotics was tested against strains that simultaneously showed more than four virulence factors and (partial) resistance to any of the antibiotics alone, a clear reduction of antimicrobial MIC (up to 100-fold) was detected. These data highlight the potency of the combinations AS-48/antibiotics to reduce the survival of strains that otherwise can easily colonize the urinary tract and are difficult to control with first-line treatments.

AS-48 is a circular, well-characterized [15,35] and safe [24] bacteriocin with a proven, broad potential in the food industry and potential health applications. This cationic and amphipathic peptide has a bactericidal effect inhibiting the growth of several pathogenic Gram-positive bacteria, such as *Staphylococcus aureus* [23], *Cutibacterium acnes* (causing cell death and impaired biofilm formation) [19], or even *Mycobacterium tuberculosis* [36], among others. In this work, the AS-48 MIC values for the uropathogenic enterococcal isolates (average 3.1 ± 1 mg/L) were similar to those required to inhibit other Gram-positive bacteria regardless of the genotypic or phenotypic characteristic of the clinical strains. This indicates, as expected, that AS-48 activity is independent of resistance to antibiotics (no cross-resistance), and the activity is not influenced by the putative expression of the virulence factors here analyzed. Cross-resistance antibiotics-bacteriocins are a broadly analyzed phenomenon, which to date seems to be ruled out due to chemical structure and the different targets (usually the lipid bilayer for many bacteriocins). Similarly to other bacteriocins [37,38,39], available data evidence the synergy of AS-48 when it was combined with other antimicrobial compounds (e.g., ethambutol, nisin, or lysozyme), leading to reductions in the MIC of both compounds [19,23,36].

Considering the potential of AS-48 in clinical settings, we explored possible combinations with well-characterized antibiotics (vancomycin, gentamicin, and amoxicillin/clavulanate), which are used as efficient antimicrobial therapies to inhibit multidrug-resistant enterococci and our results prove that in no case was there an antagonistic effect. In fact, AS-48 and the antibiotics assayed, which exhibit different mechanisms of action, showed, in general, synergistic or partially synergistic effects, regardless of the antibiotic resistance that they displayed. Thus, AS-48 MIC in combination with amoxicillin/clavulanate remained in the same order of magnitude in 2/4 cases and was reduced (10%) in the other two, whereas the MIC for the antibiotic was drastically reduced in 3/4 cases, with a synergistic effect in two of them. Interestingly, the combination of AS-48 and vancomycin usually achieved a higher sensitivity to vancomycin with 2- to 150-fold MIC reduction observed (e.g., strains 47374 and 49324). In these cases, AS-48 MIC remained almost unaffected. On the contrary, in other strains (e.g., 481467 and U-666) where the MIC of vancomycin was similar before and after the combination with AS-48, the latter was reduced 10- and 280-fold. Noticeably, the highest synergy was detected against vancomycin-resistant strains regardless of the resistance gene encoded. This finding was particularly striking in *vanA*- and *vanB*-resistant enterococci because resistance relies on the replacement of the D-Ala-D-Ala dimer by D-Ala-D-Lactate in the peptidoglycan precursor upon vancomycin induction of the *van* gene cluster. Whether AS-48 is blocking vancomycin induction of the gene cluster or the replacement between D-Ala-D-Ala by D-Ala-D-Lactate remains to be elucidated. Alternatively, it has been suggested that the introduction of positive charges in vancomycin derivatives to target the bacterial cell membrane could restore the capability of vancomycin to control even vancomycin-resistant bacteria [40]. Could this be the case with AS-48? Similarly, the mechanism behind the boost in amoxicillin sensitivity remains elusive since no direct interaction between AS-48 and penicillin-binding proteins has been demonstrated.

Finally, we examined the combination of AS-48 with gentamicin, an aminoglycoside used against enterococci in combination with other antimicrobials such as penicillin or glycopeptides due to the low permeability of the enterococcal cell membrane. In addition, genetic resistance can be acquired against this group of antibiotics [41]. The combination of AS-48, which is a membrane-permeabilizing agent, and gentamicin could be envisioned as a fruitful molecular collaboration. Although the observed effect was additive in most cases, gentamicin MIC dropped dramatically in the presence of AS-48 (up to 500-fold in almost half of the strains). We know that AS-48 permeabilizes the bacterial membrane, granting gentamicin access to the cytoplasm, although this mechanism needs further clarification.

In addition to the benefits of the simultaneous use of AS-48 and antibiotics against enterococci, the fact that AS-48 impairs biofilm formation, as it has been demonstrated with *C. acnes* [19] or *Staphylococcus aureus* (unpublished data), may promote its use as a coating agent in catheters as a prophylactic compound to control undesired growth of enterococci. Other bacteriocins in the group of lantibiotics are receiving attention due to their activity to prevent and treat biofilms [9]. In this sense, research on biocompatible, nontoxic material in catheters has tested, among others, antimicrobial peptides, including bacteriocins, obtaining promising results although clinical trials are pending [9,42,43].

## 4. Materials and Methods 

### 4.1. Isolation of Clinical Bacterial Strains and Growth Conditions

The collection of 58 enterococcal clinical strains isolated from urinary tract infections (UTIs) was initially grown on Columbia agar (BioMérieux, Madrid, Spain) added with 5% lamb blood and grown at 37 °C for 24 h. Phenotypic identification continued with Gram staining, catalase activity, and hydrolysis of esculin during growth on Bile Esculin Agar (Biomedics, Madrid, Spain). Several vancomycin-resistant strains and reference strains from the lab collection were included: *E. gallinarum* C86 (*vanC1^+^*) [44], *E. faecium* C135 (*vanB^+^*) [44], *E. faecium* AR1 (*vanA^+^*) [44], *E. casseliflavus* C85 (*vanC2^+^*) [45], *E. durans* AR23 (*vanA^+^*) [45], *E. faecalis* G8 (*vanB^+^*), *E. faecium* G74 (*vanA^+^*), *E. faecium* LMG16003 (unknown *van^+^* resistance gene), *E. faecium* LMG11423 (*van^−^*), *E. faecalis* LMG16216 (*vanB^+^*), *E. faecalis* LMG8222 (*van^−^*), and *E. faecalis* FI9190 (multiple virulence factors) [46]. MIC tests were performed in Mueller-Hinton Broth (Scharlau, Barcelona, Spain), according to internationally accepted protocols [47].

### 4.2. Genotyping and Molecular Identification

Genomic typing of the uropathogenic enterococcal population isolated was performed by randomly amplified polymorphic DNA (RAPD) followed by PCR screening of enterococcal virulence factors. Each genomic group was subsequently identified by species-specific PCR and 16S rDNA amplification and sequencing.

#### 4.2.1. RAPD

Genomic DNA was isolated by the modification of the salting-out procedure (MSOP) [48,49]. Genomic fingerprints were obtained by RAPD using the M13 primer as described previously (Supplementary Table S1) [49]. Genomic fingerprints were clustered using the Fingerprinting II software (Bio-Rad) applying Pearson product moment correlation coefficient, and the corresponding dendrogram was constructed using the unweighted-pair-group method with arithmetic averages (UPGMA) [50]. To assess the reproducibility of RAPD profiles, each PCR was performed in triplicate.

#### 4.2.2. Species Identification

Simultaneously, 16S rDNA of a representative strain of each genomic cluster was amplified with the specific primers WO1 and WO12 [51], purified, and sequenced. Next, all the strains were confirmed by species-specific PCR. To this end, the *ddl* gene (D-ala-D-ala ligase) was amplified with the primers FAC1-1 and FAC2-1 (*E. faecium*) or ddl-E1 and ddl-E2 (*E. faecalis*) [52]. All the primers used in this work are listed in Supplementary Table S4.

### 4.3. Purification of the Bacteriocin AS-48

AS-48 from concentrated supernatants of the producer strain *E. faecalis* UGRA10 was purified to homogeneity (up to 95% purity) by cation exchange (Sepharose BigBeads) (GE Healthcare, Madrid, Spain), followed by reversed-phase, high-performance liquid chromatography (RP-HPLC) and monitoring using antimicrobial activity tests as described elsewhere [24]. The purified samples were aliquoted and freeze-dried for storage at 4 °C under vacuum. When required, it was solubilized in MilliQ water and the concentration was measured spectrophotometrically, applying the molecular extinction coefficient following standard methods [53].

### 4.4. Determination of the Minimal Inhibitory Concentration (MIC)

A preliminary sensitivity test using the WIDER I system was used to check the antibiotic susceptibility of the clinical isolates [54]. Additionally, MIC determination was carried out by broth microdilution assay following the adapted Clinical and Laboratory Standards Institute (CLSI) indications [47]. Stocks of HPLC-purified AS-48 (0.3 mg/mL) and the antibiotics vancomycin (50 mg/mL) and amoxicillin/clavulanic acid (43.7/6.2 mg/mL) were prepared separately by dissolving them in MilliQ water. The stock solutions were filter-sterilized and aliquoted in single-use vials for storage at −20 °C. Gentamicin was purchased as a sterile solution (80 mg/mL), aliquoted, and stored at −20 °C. The starting concentration in the microtiter plate for the MIC tests was 500 μg/mL for vancomycin, 128 μg/mL for gentamicin, 10.9/1.55 μg/mL for amoxicillin/clavulanate, and 16.8 μg/mL for AS-48.

### 4.5. Determination of the Synergy between AS-48 and Antibiotics

Synergistic analysis for the combination of AS-48 and antibiotics (vancomycin, gentamicin, and amoxicillin/clavulanate) was performed against the selected strains, for which resistance to these antibiotics was higher. The combined effects of AS-48 and antibiotic was evaluated in triplicate from the fractional inhibitory concentration index (FICI) for each combination in 96-well microtiter plates, using the microdilution checkerboard test and according to the CLSI recommendations [55]. The FICI results for each combination against each isolate were interpreted as follows: The synergistic effect was defined as FICI ≤ 0.5, partial synergism as 0.5 < FICI < 1, additivity as FICI = 1, indifference as 1 < FICI ≤ 4, and antagonism as FICI > 4 [55].

## 5. Conclusions

In summary, our results prove the potent effect of the bacteriocin AS-48 against a set of clinical uropathogenic enterococcal strains. The antimicrobial combinatorial therapy of AS-48 and antibiotics, with distinct mechanisms of action, is of particular interest in the case of multidrug-resistant strains, which remain sensitive to AS-48. Such combinations lead to additive or synergistic effects and result in reduction of the concentration of drugs against these bacteria. Similar synergistic behavior has been observed with other bacteriocins used in combination with antibiotics against clinical and veterinary pathogens [39]. These data, together with the safety of AS-48, lacking of toxic effects demonstrated in in vivo and in vivo assays [24], strongly support this bacteriocin as a leading compound to fight enterococcal infections. Moreover, its mechanism of action reduces the appearance of resistance. Our results also prove that exploiting combined antibiotic therapy with antimicrobial peptides, such as AS-48, is an alternative of broad interest to introduce new drugs to fight against multi-resistant bacteria [12,56].

## Figures and Tables

**Table 1:** Distribution of the virulence factors between the clinical isolates; *gelE*, gelatinase; *hyl*, hyaluronidase; *asa1*, aggregation substance; *cylA*, cytolysin A; *esp*, extracellular surface protein; *ace*, accessory colonization factor; *efaA*, adhesin.

Genotype	No. Strains	% of Strains	Total % of Strains
*gelE*	1	1.72	6.89 (1) ^a^
*hyl*	1	1.72	
*asa1*	1	1.72	
*efaA*	1	1.72	
*gelE + ace*	1	1.72	19.96 (2) ^a^
*gelE + efaA*	6	10.34	
*asa1 + esp*	3	5.17	
*esp + efaA*	1	1.72	
*gelE + hyl + efaA*	1	1.72	27.58 (3) ^a^
*gelE + asa1 + efaA*	9	15.52	
*gelE + esp + efaA*	2	3.45	
*gelE + ace + efaA*	2	3.45	
*asa1 + esp + efaA*	1	1.72	
*asa1 + ace + efaA*	1	1.72	
*gelE + asa1 + esp + efaA*	6	10.34	27.58 (4) ^a^
*gelE + asa1 + cylA + efaA*	2	3.45	
*gelE + asa1 + ace + efA*	5	8.62	
*gelE + esp + ace + efaA*	2	3.45	
*asa1 + cylA + ace + efaA*	1	1.72	
*asa1 + esp + cy l+ ace + efaA*	1	1.72	13.79 (5) ^a^
*gelE + asa1 + esp + ace + efaA*	5	8.62	
*gelE + asa1 + cylA + ace + efaA*	2	3.45	
*gelE + asa1 + esp + cylA + ace + efaA*	3	5.17	5.17 (6) ^a^

^a^ Number of co-occurrent virulence factors.

**Table 2:** MIC (in mg/L) of lab enterococcal strains in relation to the average of the clinical isolates for AS-48, vancomycin (Van), gentamicin (Gent), and amoxicillin/clavulanate (Amo/Cla).

	MIC (mg/L)			
	AS-48	Van	Gent	Amo/Cla
*E. gallinarum* C86	10	7.8	1.5	2.7
*E. casseliflavus* C85	10	1.9	0.5	0.3
*E. durans* AR23	8.7	>500	2	0.7
*E. faecium* C135	8.7	20.8	>128	0.3
*E. faecium* AR1	4.4	>500	5	10.9
*E. faecium* G8	14	>500	4	2.7
*E. faecium* LMG11423	21	1.6	8	1.4
*E. faecalis* LMG08222	5.2	3.9	4	1.4
*E. faecalis* LMG16216	21	12	>128	0.7
*E. faecalis* LMG16003	7	125	6	1.4
*E. faecalis* G74	10.5	31.3	>128	0.7

**Table 3:** Combination of AS-48 and vancomycin against a selection of clinical and lab enterococci strains.

Strain	Van^R^ Gen	Van (1)	AS-48 (1)	Van (2)	AS-48 (2)	FICI
U-666	-	3.91	5.67	4.7	0.02	1.21
U-1055	-	3.91	3.78	3.9	4.25	2.12
U-1641	-	3.91	3.78	3.9	4.25	2.12
U-230	-	3.91	2.83	1.95	4.25	2
481467	-	7.81	2.83	3.9	0.27	0.59
47374	-	4.56	3.54	0.03	1.05	0.3
481413	-	2.6	4.25	0.03	2.1	0.51
49324	-	3.91	2.83	0.03	2.1	0.75
49768	-	3.91	2.83	3.9	1.45	1.51
49785	-	2.6	4.25	0.03	0.5	0.13
49890	-	2.6	4.25	0.03	0.5	0.13
491698	-	3.26	7.08	0.03	1	0.15
*E. gallinarum* C86	*vanC1*	7.8	10	0.97	1.3	0.25
*E. casseliflavus* C85	*vanC2*	1.9	10	0.48	0.16	0.27
*E. durans* AR23	*vanA*	>500	8.7	62.5	1.3	<0.27 *
*E. faecium* C135	*vanB*	20.8	8.7	1.9	0.02	0.09
*E. faecium* AR1	*vanA*	>500	4.4	250	0.65	<0.65 *
*E. faecium* G8	*vanA*	>500	14	31	0.32	<0.08 *
*E. faecium* LMG11423	ND	1.6	21	0.12	0.32	0.09
*E. faecalis* G74	*vanB*	31.3	10.5	15.1	0.32	0.51
*E. faecalis* LMG08222	ND	3.9	5.2	0.24	0.16	0.09
*E. faecalis* LMG16216	*vanB*	12	21	0.97	0.65	0.11
*E. faecalis* LMG16003	ND	125	7	7.8	0.04	0.07

Van (1), MIC for vancomycin alone; AS-48 (1), MIC for AS-48 alone; Van (2), MIC for vancomycin in the combination; AS-48 (2), MIC for AS-48 in the combination. In green, synergistic combinations; in orange, additive effect. In grey, four-fold or higher reduction in the MIC observed. ND: Not determined. * In these cases, the calculation was done considering that the MIC for the individual compound was exactly 500 mg/L.

**Table 4:** Combination of AS-48 and gentamicin against a selection of clinical and lab enterococci strains.

Strains	Gent (1)	AS-48 (1)	Gent (2)	AS-48 (2)	FICI
U-1688	128	3.15	0.25	3.15	1
U-1593	128	1.9	0.25	1.9	1
U-1641	128	3.8	0.25	3.8	1
U-230	128	2.83	0.5	2.83	1
U-1839	128	4.25	0.25	4.25	1
U-814	128	1.89	128	1.89	2
U-1846	128	1.89	64	1.89	1.51
481609	128	4.25	32	1.06	0.5
491284	128	2.5	0.25	2.5	1
49785	128	4.25	16	1.15	0.4
*E. faecium* C135	128	20.8	8	2.6	0.19
*E. faecalis* G74	128	31.3	0.25	3.9	0.13
*E. faecalis* LMG16216	128	21	2	10.5	0.52

Gent (1), MIC for gentamicin alone; Gent (2), MIC for gentamicin in the combination; AS-48 (1), MIC for AS-48 alone; AS-48 (2), MIC for AS-48 in the combination. In green, synergistic combinations; in orange, additive effect. In grey, four-fold or more reduction in the MIC observed.

**Table 5:** Combination of AS-48 and amoxicillin/clavulanate synergy tests against four enterococci strains.

Strain	Amo/Cla (1)	AS-48 (1)	Amo/Cla (2)	AS-48 (2)	FICI
Sb-7	10.9	1.9	0.01	1.9	1
47391	10.93	3.54	5.47	3.5	1.49
491802	136.71	2.12	17	0.25	0.24
*E. faecium* AR1	10.9	4.4	0.09	0.34	0.09

Amo/Cla (1), MIC for amoxicillin/clavulanate alone; Amo/Cla (2), MIC for amoxicillin/clavulanate in the combination; AS-48 (1), MIC for AS-48 alone; AS-48 (2), MIC for AS-48 in the combination. In green, synergistic combinations; in orange, additive effect. In grey, four-fold or more reduction in the MIC.

## Supplementary Materials

The following are available online at https://www.mdpi.com/2079-6382/9/9/567/s1, Supplementary Tables S1–S4. Table S1: Genotypic characterization of the isolated clinical strains, Table S2: Phenotypic antibiotic resistance characterization using a panel Wider I against 20 antibiotics, Table S3: MIC (mg/L) for AS-48, vancomycin (Van), gentamicin (Gen), and amoxicillin/clavulanate (Amo/Cla), Table S4: Primers and PCR conditions used in this work.
