# Supplementary material for: Synergy of the Bacteriocin AS-48 and Antibiotics against Uropathogenic Enterococci
Source: Antibiotics (Basel). 2020 Sep 2;9(9):567. doi: 10.3390/antibiotics9090567 (PMC7558097; doi:10.3390/antibiotics9090567)
Supplement: Supplementary file 1 [file antibiotics-09-00567-s001.pdf]

Supplementary Materials

Table S1. Genotypic characterization of the isolated clinical strains. Ef, *Enterococcus faecium*, EF, *E. faecalis. gel*, gelatinase; *hyl*, hyaluronidase; *asa1*, aggregation substance; *cylA*, cytolysin A; *esp*, extracellular surface protein; *ace*, accessory colonization factor; *efaA*, adhesin*; vanA/B*, vancomycin resistance genes.

**Genotypic characterization**											
***Enterococcus* strains**	**RAPD group**	***Enterococcus* spp.**	***gelE***	***hyl***	***asa1***	***esp***	***cylA***	***ace***	***efaA***	***vanA***	***vanB***
Sb7	G1	Ef	-	-	+	+	-	-	-	-	-
47391	G1	Ef	-	-	+	+	-	-	-	-	-
491802	G2	Ef	-	+	-	-	-	-	-	-	-
49767	G3	EF	+	-	-	+	-	-	+	-	-
49867	G3	EF	+	-	-	+	-	+	+	-	-
49890	G3	EF	+	-	+	-	-	+	+	-	-
491047	G3	EF	+	-	-	-	-	-	+	-	-
491698	G3	EF	+	-	-	-	-	-	+	-	-
U-666	G4	EF	+	-	+	+	-	-	+	-	-
U-603	G4	EF	+	-	+	+	-	-	+	-	-
U-1688	G4	EF	+	-	+	+	-	-	+	-	-
U-230	G4	EF	+	-	+	+	-	-	+	-	-
481467	G4	EF	+	-	+	+	-	-	+	-	-
U-134	G4	EF	+	-	+	-	-	-	+	-	-
U-1593	G4	EF	+	-	+	-	-	-	+	-	-
491051	G4	EF	+	-	+	-	-	-	+	-	-
491044	G4	EF	+	-	+	-	-	-	+	-	-
U-1739	G4	EF	+	-	+	-	-	-	+	-	-
U-1683	G4	EF	+	-	+	-	-	-	+	-	-
U-1261	G4	EF	+	-	-	-	-	-	+	-	-
U-834	G4	EF	+	-	-	-	-	-	+	-	-
U-1641	G4	EF	+	-	-	-	-	-	+	-	-
U-1822	G4	EF	+	-	-	+	-	-	+	-	-
U-1839	G4	EF	-	-	+	+	-	-	+	-	-
U-814	G4	EF	-	-	+	+	-	-	-	-	-
U-1564	G4	EF	-	-	+	-	-	-	-	-	-
481396	G4	EF	-	-	+	-	+	+	+	-	-
4953	G4	EF	+	-	+	-	+	+		-	-
49768	G4	EF	+	-	-	-	-	+	+	-	-

491284	G4	EF	+	-	+	+	-	+	+	-	-
49891	G4	EF	+	-	-	-	-	+	-	-	-
47374	G4	EF	+	-	+	+	+	+	+	-	-
481398	G5	EF	+	-	+	-	-	+	+	-	-
U-800	G5	EF	+	-	-	-	-	-	+	-	-
481609	G5	EF	+	-	-	-	-	-	+	-	-
481413	G5	EF	+	-	+	-	-	+	+	-	-
49850	G5	EF	+	-	-	-	-	+	+	-	-
49454	G6	EF	-	-	+	+	+	+	+	-	-
U-1846	G6	EF	+	-	+	-	-	-	+	-	-
U-1679	G7	EF	-	-	-	-	-	-	+	-	-
49353	G7	EF	+	+	-	-	-	-	+	-	-
491440	G7	EF	+	-	+	-	+	+	+	-	-
491797	G7	EF	+	-	+	-	-	-	+	-	-
491376	G8	EF	+	-	+	-	-	+	+	-	-
49324	G8	EF	+	-	+	-	-	+	+	-	-
U-1765	G9	EF	-	-	-	+	-	-	+	-	-
47342	G9	EF	-	-	+	-	-	+	+	-	-
491336	G9	EF	+	-	+	-	+	-	+	-	-
U-638	G9	EF	+	-	+	+	-	-	+	-	-
U-1055	G9	EF	+	-	+	+	-	+	+	-	-
U-80	G9	EF	+	-	-	-	-	-	-	-	-
4784	G9	EF	+	-	+	+	+	+	+	-	-
491612	G9	EF	+	-	-	+	-	+	+	-	-
491209	G9	EF	+	-	+	+	-	+	+	-	-
4110PR-94	G9	EF	+	-	+	+	+	+	+	-	-
47376	G10	EF	+	-	+	+	-	+	+	-	-
491049	G11	EF	+	-	+	-	-	-	+	-	-
49785	G12	EF	+	-	+	-	+	-	+	-	-
		Positive (%)	81.03	3.45	67.24	39.66	13.79	37.93	87.93	0	0
		Negative (%)	18.97	96.55	32.76	60.34	86.21	62.07	12.07	100	100

Table S2: Phenotypic antibiotic resistance characterization using a panel Wider I against 20 antibiotics. 1: Penicillin; 2: Ampicillin; 3: Amoxicillin/Clavulanate; 4: Oxacillin; 5: Cefazolin; 6: Cefotaxime; 7: Streptomycin 1000; 8: Gentamicin 500; 9: Amikacin; 10: Vancomycin; 11: Teicoplanin; 12: Levofloxacin; 13: Erythromycin; 14: Clindamycin; 15: Quinupristin/Dalfopristin; 16: Linezolid; 17: Chloramphenicol; 18: Fosfomycin; 19: Trimethoprim/sulfamethoxazole; 20: Rifampicin.

	**Antibiotic resistance profile Wider I panel**																			
***Enterococcus* strains**	1	2	3	4	5	6	7	8	9	10	11	12	13	14	15	16	17	18	19	20
Sb7	R	R	R	R	R	R	R	S	R	S	S	R	R	R	R	S	S	S	R	R
47391	R	R	R	R	R	R	R	S	R	S	S	R	R	R	R	S	S	S	R	I
491802	R	R	R	R	R	R	S	S	R	S	S	R	R	R	S	S	S	S	R	S
49767	S	S	S	R	R	R	R	S	R	S	S	S	R	R	R	S	R	S	R	R
49867	S	S	S	R	R	R	S	S	R	S	S	S	I	R	R	S	S	S	R	I
49890	S	S	S	R	R	R	S	S	R	S	S	S	R	R	R	S	S	S	R	I
491047	S	S	S	R	R	R	S	S	R	S	S	R	R	R	R	S	S	S	R	R
491698	S	S	S	R	R	R	R	S	R	S	S	S	I	R	R	S	S	S	R	I
U-666	S	S	S	R	R	R	S	S	R	S	S	S	S	R	R	S	S	S	R	S
U-603	S	S	S	R	R	R	S	R	R	S	S	S	R	R	R	S	S	S	R	R
U-1688	S	S	S	R	R	R	R	R	R	S	S	I	R	R	R	S	S	S	R	R
U-230	S	S	S	R	R	R	R	S	R	S	S	R	R	R	R	S	S	S	R	I
481467	S	S	S	R	R	R	S	S	R	S	S	S	R	R	R	S	S	S	R	I
U-134	S	S	S	R	R	R	R	R	R	S	S	S	R	R	R	S	S	S	R	S
U-1593	S	S	S	R	R	R	R	R	R	S	S	I	R	R	R	S	R	S	R	I
491051	S	S	S	R	R	R	R	S	R	S	S	R	R	R	R	S	S	S	R	I
491044	S	S	S	R	R	R	S	S	R	S	S	R	S	R	R	S	S	R	R	I
U-1739	S	S	S	R	R	R	S	S	R	S	S	S	S	R	R	S	S	S	R	R
U-1683	S	S	S	R	R	R	R	S	R	S	S	S	R	R	R	S	R	S	R	I
U-1261	S	S	S	R	R	R	S	S	R	S	I	S	R	R	R	S	S	S	R	R
U-834	S	S	S	R	R	R	S	S	R	S	S	S	S	R	R	S	S	S	R	R
U-1641	S	S	S	R	R	R	R	R	R	S	S	S	R	R	R	S	R	S	R	R
U-1822	S	S	S	R	R	R	R	R	R	S	S	S	I	R	R	S	S	S	R	R
U-1839	S	S	S	R	R	R	R	R	S	S	S	S	R	R	R	S	S	S	R	R
U-814	S	S	S	R	R	R	R	R	R	S	S	S	R	R	R	S	S	S	R	R
U-1564	S	S	S	R	R	R	S	S	R	S	S	S	I	R	R	S	S	S	R	I
481396	S	S	S	R	R	R	S	S	R	S	S	S	R	R	R	S	S	S	R	S
4953	S	S	S	R	R	R	R	R	R	S	S	S	R	R	R	S	R	R	R	I
49768	S	S	S	R	R	R	S	S	R	S	S	R	S	R	R	S	S	S	R	I
491284	S	S	S	R	R	R	R	R	R	S	S	R	R	R	R	S	S	S	R	I
49891	S	S	S	R	R	R	S	S	R	S	S	R	R	R	R	S	S	S	R	I
47374	S	S	S	R	R	R	R	S	R	S	S	I	S	R	R	S	S	S	R	I
481398	S	S	S	R	R	R	S	S	R	S	S	S	I	R	R	S	S	S	R	I
U-800	S	S	S	R	R	R	R	S	R	S	S	R	R	R	R	S	R	S	R	I
481609	S	S	S	R	R	R	R	S	R	S	S	R	R	R	R	S	R	S	R	I
481413	S	S	S	R	R	R	S	S	R	S	S	S	R	R	R	S	R	S	R	I

49850	S	S	S	R	R	R	R	S	R	S	S	S	R	R	R	S	S	S	R	R
49454	S	S	S	R	R	R	R	S	R	S	S	S	R	R	R	S	S	S	R	I
U-1846	S	S	S	R	R	R	S	S	R	S	S	S	R	R	R	S	S	S	R	S
U-1679	S	S	S	R	R	R	S	S	R	S	S	S	S	R	R	S	S	S	R	S
49353	S	S	S	R	R	R	S	S	R	S	S	R	R	R	R	S	S	S	R	I
491440	S	S	S	R	R	R	S	S	R	S	S	S	I	R	R	S	S	S	R	I
491797	S	S	S	R	R	R	S	S	R	S	S	S	S	R	R	S	S	S	R	I
491376	S	S	S	R	R	R	S	S	R	S	S	S	S	R	R	S	S	S	R	I
49324	S	S	S	R	R	R	S	S	R	S	S	S	I	R	R	S	S	S	R	I
U-1765	S	S	S	R	R	R	R	S	R	S	S	S	R	R	R	S	R	S	R	R
47342	S	S	S	R	R	R	R	S	R	S	S	S	R	R	R	S	S	S	R	R
491336	S	S	S	R	R	R	R	S	R	S	S	S	R	R	R	S	S	S	R	I
U-638	S	S	S	R	R	R	S	S	R	S	S	S	I	R	R	S	S	S	R	I
U-1055	S	S	S	R	R	R	S	S	R	S	S	S	R	R	S	S	S	S	R	I
U-80	S	S	S	R	R	R	S	R	R	S	S	S	R	R	R	S	R	S	R	R
4784	S	S	S	R	R	R	R	S	R	S	S	S	R	R	R	S	R	S	R	I
491612	S	S	S	R	R	R	S	S	R	S	S	S	R	R	R	S	S	S	R	I
491209	S	S	S	R	R	R	S	S	R	S	S	S	R	R	R	S	R	S	R	I
4110PR-94	S	S	S	R	R	R	R	S	R	S	S	S	R	R	R	S	S	S	R	I
47376	S	S	S	R	R	R	S	S	R	S	S	S	I	R	R	S	S	S	R	R
491049	S	S	S	R	R	R	S	S	R	S	S	S	I	R	R	S	S	S	R	I
49785	S	S	S	R	R	R	R	R	R	S	S	S	R	R	R	S	S	S	R	I
Resistant %	5.2	5.2	5.2	100	100	100	46.6	20.7	98.3	0	0	22.4	67.2	100	96.6	0	20.7	3.4	100	29.3
Sensitive %	94.8	94.8	94.8	0	0	0	53.4	79.3	1.7	100	98.3	72.4	15.5	0	3.4	100	79.3	96.6	0	10.3
Intermedial %	0	0	0	0	0	0	0	0	0	0	1.7	5.2	17.2	0	0	0	0	0	0	60.3

Table S3: MIC (mg/L) for AS-48, vancomycin (Van), gentamicin (Gen) and amoxicillin/clavulanate (Amo/Cla).

	MIC (mg/ L)			
***Enterococcus* strains**	**AS-48**	**Van**	**Gen**	**Amo/Cla**
Sb7	1.9	1.6	4	10.9
47391	3.5	0.9	8	10.9
491802	2.1	1.9	8	136.7
49767	2.1	2.6	16	1.4
49867	4.2	1.9	16	0.7
49890	4.2	2.6	16	0.7
491047	2.8	1.9	16	0.7
491698	7.1	3.2	64	0.3
U-666	5.6	3.9	16	1.4
U-603	3.8	2.6	16	0.7
U-1688	3.1	1.9	>128	1.4
U-230	2.8	3.9	>128	1.4
481467	2.8	7.8	16	0.3
U-134	3.8	1.9	16	0.7
U-1593	1.9	3.9	>128	0.7
491051	2.8	3.2	16	0.2
491044	3.5	3.9	16	1.4
U-1739	3.1	2.6	16	0.2
U-1683	2.5	3.2	32	0.2
U-1261	3.5	3.2	32	0.7
U-834	1.9	2.6	16	0.7
U-1641	3.8	3.9	>128	0.7
U-1822	2.1	1.9	16	1.4
U-1839	2.5	3.2	128	1.4
U-814	1.9	1.9	>128	0.5
U-1564	1.9	1.9	32	0.2
481396	2.8	3.9	16	0.7
4953	2.8	1.9	64	1.4
49768	2.8	3.9	32	1.4
491284	4.2	3.9	>128	1.4
49891	2.1	2.6	16	0.7

47374	3.5	4.5	16	0.3
481398	4.2	3.2	16	1.4
U-800	1.9	2.6	16	0.7
481609	4.2	1.9	>128	0.7
481413	4.2	2.6	4	1.4
49850	4.2	3.2	16	1.4
49454	2.1	1.9	16	1.4
U-1846	1.3	2.6	>128	0.2
U-1679	3.5	3.9	16	1.4
49353	2.1	1.9	16	0.06
491440	3.5	3.9	16	0.2
491797	2.1	3.9	16	0.2
491376	2.1	3.9	16	0.3
49324	2.8	3.9	16	1.4
U-1765	2.5	2.6	8	0.7
47342	4.2	2.9	16	1.4
491336	3.5	1.9	2	0.3
U-638	3.6	1.9	8	0.2
U-1055	3.8	2.6	8	0.3
U-80	3.8	1.9	16	1.4
4784	3.5	3.9	32	0.7
491612	3.5	1.9	32	1.4
491209	2.1	3.9	16	1.4
4110PR-94	2.1	3.9	16	0.7
47376	3.5	1.3	16	0.06
491049	2.8	1.9	16	0.7
49785	4.2	2.6	>128	1.4
Average±stdeva	3.1±1	2.9±1.1	37.3±43.2	3.5±17.9

Table S4: Primers and PCR conditions used in this work.

**Primer**	**Sequence 5'-3'**	**Purpose**	**PCR conditions**	**Reference**
M13	gagggtggcggttct	RAPD	1 × 94 °C 1 m, 35 × (94 °C 1 m, 40 °C 20 s, 72 °C 1:20 m) 1 × 72 °C 5 m.	[1]
Entero 1	cccggctcaaccggg	*Enterococcus* 16S rDNA	1 × 94 °C 1 m, 30 × (94 °C 1 m, 60 °C 1 m, 72 °C 1 m) 1 × 72 °C 5 m.	[2]
Entero 2	ctctagagtggtcaa			
FAC1-1	gagtaaatcactgaacga	*E. faecium ddl* gene	1 × 94 °C 3 m, 30 × (94 °C 1 m, 54 °C 1 m, 72 °C 1 m) 1 × 72 °C 7 m.	[3]
FAC2-1	cgctgatggtatcgattcat			
ddl-E1	atcaagtacagttagtctt	*E. faecalis ddl* gene		[4]
ddl-E2	acgattcaaagctaactg			
Van-Af	tctgcaatagagatagccgc	*vanA*	1 × 94 °C 3 m, 30 × (94 °C 1 m, 55 °C 1 m, 72 °C 1 m) 1 × 72 °C 5 m.	[5]
Van-Ar	ggagtagctatcccagcatt			
Van-Bf	gctccgcagcctgcatggaca	*vanB*		
Van-Br	acgatgccgccatcctcctgc			
CYT I	actcggggattgataggc	*cylA*		[6]
CYT IIb	gctgctaaagctgcgctt			
Gel11_for	tatgacaatgctttttgggat	*gelE*		
Gel12_rev	agatgcacccgaaataatata			
*ASA1*1_for	gcacgctattacgaactatga	*asa1*		
*ASA1*2_rev	taagaaagaacatcaccacga			
*Hyl*1_for	acagaagagctgcaggaaatg	*hyl*		
*Hyl*12_rev	gactgacgtccaagtttccaa			
Esp14F_for	agatttcatctttgattcttgg	*esp*		
Esp12R_rev	aattgattctttagcatctgg			
EFA-AF	gccaattgggacagaccctc	*efaA*		[7,8]
EFA-AR	cgccttctgttccttctttggc			
ACE-F	gaattgagcaaaagttcaatcg	*ace*		
ACE-R	gtctgtcttttcacttgtttc			

References:

1. Martín-Platero, A.M.; Maqueda, M.; Valdivia, E.; Purswani, J.; Martínez-Bueno, M. Polyphasic study of microbial communities of two Spanish farmhouse goats' milk cheeses from Sierra de Aracena. *Food Microbiol.* **2009**, *26,* 294–304, doi:10.1016/j.fm.2008.12.004.
2. Deasy, B.M.; Rea, M.C.; Fitzgerald, G.F.; Cogan, T.M.; Beresford, T.P. A rapid PCR based method to distinguish between *Lactococcus* and *Enterococcus*. *Syst. Appl. Microbiol.* **2000**, *23,* 510–522, doi:10.1016/S0723-2020(00)80025-9.
3. Depardieu, F.; Perichon, B.; Courvalin, P. Detection of the *van* alphabet and identification of Enterococci and Staphylococci at the species level by multiplex PCR. *J. Clin. Microbiol.* **2004**, *42*, 5857–5860, doi:10.1128/JCM.42.12.5857-5860.2004.

4. Dutka-Malen, S.; Evers, S.; Courvalin, P. Detection of glycopeptide resistance genotypes and identification to the species level of clinically relevant enterococci by PCR. *J. Clin. Microbiol.* **1995**, *33*, 1434.
5. Lemcke, R.; Bülte, M. Occurrence of the vancomycin-resistant genes *vanA*, *vanB*, *vanC1*, *vanC2* and *vanC3* in *Enterococcus strains* isolated from poultry and pork. *Int. J. Food Microbiol.* **2000**, *60*, 185–194, doi:10.1016/S0168-1605(00)00310-X.
6. Vankerckhoven, V.; Van Autgaerden, T.; Vael, C.; Lammens, C.; Chapelle, S.; Rossi, R.; Jabes, D.; Goossens, H. Development of a multiplex PCR for the detection of *asa1*, *gelE*, *cylA*, *esp*, and *hyl* genes in enterococci and survey for virulence determinants among European hospital isolates of *Enterococcus faecium*. *J. Clin. Microbiol.* **2004**, *42*, 4473–4479, doi:10.1128/JCM.42.10.4473-4479.2004.
7. Ben Omar, N.; Castro, A.; Lucas, R.; Abriouel, H.; Yousif, N.M.K.; Franz, C.M.A.P.; Holzapfel, W.H.; Pérez-Pulido, R.; Martínez-Cañamero, M.; Gálvez, A. Functional and safety aspects of Enterococci isolated from different Spanish foods. *Syst. Appl. Microbiol.* **2004**, *27*, 118–130, doi:10.1078/0723-2020-00248.
8. Creti, R.; Imperi, M.; Bertuccini, L.; Fabretti, F.; Orefici, G.; Di Rosa, R.; Baldassarri, L. Survey for virulence determinants among *Enterococcus faecalis* isolated from different sources. *J. Med. Microbiol.* **2004**, *53*, 13–20, doi:10.1099/jmm.0.05353-0.
